# Cognition and Evolution of Movement Disorders of *FOXG1*-Related Syndrome

**DOI:** 10.3389/fneur.2019.00641

**Published:** 2019-06-28

**Authors:** Lee-Chin Wong, Yen-Tzu Wu, Chia-Jui Hsu, Wen-Chin Weng, Wen-Che Tsai, Wang-Tso Lee

**Affiliations:** ^1^Department of Pediatrics, Cathay General Hospital, Taipei, Taiwan; ^2^Graduate Institute of Clinical Medicine, National Taiwan University College of Medicine, Taipei, Taiwan; ^3^School and Graduate Institute of Physical Therapy, National Taiwan University College of Medicine, Taipei, Taiwan; ^4^Department of Physical Medicine and Rehabilitation, National Taiwan University, Taipei, Taiwan; ^5^Department of Pediatrics, Taipei City Hospital YangMing Branch, Taipei, Taiwan; ^6^Department of Pediatrics, National Taiwan University Hospital, Taipei, Taiwan; ^7^Department of Psychiatry, National Taiwan University Hospital, Taipei, Taiwan; ^8^Graduate Institute of Brain and Mind Sciences, National Taiwan University College of Medicine, Taipei, Taiwan

**Keywords:** *FOXG1*, movement disorder, hyperkinetic, hypokinetic, evolution, cognition

## Abstract

*FOXG1*-related syndrome is a rare neurodevelopmental encephalopathy characterized by early onset hyperkinetic movement disorders, absent language, autistic features, epilepsy, and severe cognitive impairment. However, detailed evaluation of cognition and evolution of movement disorders over time have not been clearly described before. In this study, we performed whole-exome sequencing in a cohort with unknown severe encephalopathy and movement disorders, with/without autistic behaviors. We identified *FOXG1* mutations in three patients. One of them had a novel mutation that has not been described before. The neuropsychological test by Mullen Scales of Early Learning (MSEL) showed severe psychomotor impairments in all patients. There were uneven cognitive abilities in terms of verbal and non-verbal cognitive domains in all of them, with approximately 2 months differences. Gross motor skills and expressive language were more severely affected than the other domains in all the patients. All individuals had early onset hyperkinetic movement disorders. The movement disorders in one of our patients changed from predominantly hyperkinetic in early childhood to more hypokinetic in adolescence with the development of dystonia. To the best of our knowledge, this evolution had never been described before. In conclusion, individuals with *FOXG1*-related syndrome may show clinical progression from hyperkinetic to hypokinetic features over time. There were also uneven cognitive abilities in verbal and non-verbal cognitive domains. The *FOXG1* mutation should be considered in individuals with a history of hyperkinetic movements, microcephaly, and uneven cognitive abilities with characteristic brain images.

## Introduction

*FOXG1*-related syndrome is a rare neurodevelopmental encephalopathy, associated with heterozygous variants in the forkhead box G1 *(FOXG1)* gene. Although this syndrome was initially described as a congenital Rett syndrome (RTT) variant (1–4), children with this syndrome have clinical presentations and cerebral malformations distinguishable from those in RTT. This syndrome is characterized by developmental delays, autism-like traits, microcephaly, absence of language, severe cognitive disabilities, early-onset dyskinesia, stereotypic hand movements, epilepsy, and corpus callosum dysgenesis (5, 6). Fewer than 90 cases have been reported worldwide to date (7–9), and the disease is also very rare in Taiwan (10).

Early-onset hyperkinetic movement disorders, such as choreoathetosis and orolingual/facial dyskinesias, which are usually non-responsive to medication, are the hallmarks of this disease (7, 11). However, progression of the movement disorders overtime has not been reported. In addition, although developmental delays are a common feature, few studies have conducted psychological evaluations on these patients (7). The evaluation of cognitive abilities in children with *FOXG1*-related syndrome is challenging because of their limited language ability that resembles that of children with autism spectrum disorder (ASD) (12). The comprehensive norm-referenced developmental test called Mullen Scales of Early Learning (MSEL) (13) is widely used for the assessment of young children with ASD or developmental disabilities (14, 15). Since many behavioral features can further interfere with the accurate assessment of children's cognitive abilities, the MSEL can be used to quickly and easily test young children with autism-like traits, and it identifies the strengths and weaknesses in verbal and non-verbal skills of these children with limited social interaction and communication skills (16).

Herein, three cases with *FOXG1*-related syndrome were found by whole-exome sequencing, and we describe their clinical characteristics and progression. To the best of our knowledge, this is the first report on *FOXG1*-related syndrome from Han Chinese children in Taiwan.

## Methods and Materials

### Patient Enrollment

Children or infants with unknown developmental delay, movement disorders, autistic behavior, and severe encephalopathy without definite diagnosis were enrolled for analysis in the present study. Molecular diagnosis was done by whole exome sequence in those suspected to have genetic etiologies. The clinical presentations, variation of movement disorders, and evolution of clinical symptoms were then analyzed. The study had been approved by the Ethical Committee of National Taiwan University Hospital, and written informed consents were obtained from the parents of all subjects.

### Whole Exome Sequence

Whole-exome sequencing was performed in all samples. Illumina VariantStudio 3.0 software was used for annotation of detected variants. Sanger sequencing was used to confirm the results of whole exome sequence.

### Neuropsychological Evaluation

MSEL, a comprehensive norm-referenced developmental test for children aged from 0 to 68 months, was used (17). The instrument contains 5 subscales: Visual Reception, Expressive Language, Receptive Language, Gross Motor, and Fine Motor scales. A non-verbal mental age was constructed for each child via averaging the age equivalents from the visual reception and fine motor scales. A verbal mental age was constructed for each child by averaging the age equivalents from the Receptive Language and Expressive Language scales.

A development quotient (DQ) is an alternative and numerical indicator to help illustrate the maturity of child's cognitive ability. The overall DQ, summed by the DQ of 4 MSEL subscales (i.e., Visual Reception, Fine Motor, Receptive Language, Expressive Language scales), was derived from the MSEL as representative of overall cognitive function. The DQ is comparable with intellectual quotient (IQ) with DQ ≧ 85 considered normal development (18–20). The MSEL has been reported to have good reliability and moderate to high correlations with the Bayley Mental Development Index and the Peabody Fine Motor Scale (17). In addition, previous studies have found that the MSEL has acceptable discriminative validity in that children with ASD obtain lower MSEL scores than typically-developing children (15, 21, 22) or children with global developmental delay (22).

## Results

**Figure 1:**
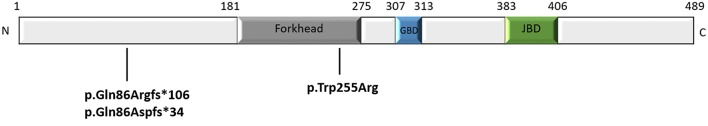
Schematic representation of the FOXG1 protein (consisting of 489 amino acid) and the mutations of our patients. The N-terminal domain, fork-head domain, the Groucho-binding domain (GBD), the JARID1B binding domain (JBD), and C-terminal domain of FOXG1 are shown.

We performed whole-exome sequencing in samples from a total of 30 cases with unknown severe encephalopathy and movement disorders, with/without autistic behaviors. We found **three** children with *FOXG1*-related syndrome (Table 1). Case 1 had a missense mutation c.763T>C (p.Trp255Arg), located in the forkhead domain; cases 2 and 3 had altered base pairs of 256 in the N-terminal domain, and both harbored c.256delC (p.Gln86Argfs^*^106) and c.256dupC (p.Gln86Aspfs^*^34) that generated truncated proteins completely or partially lacking the forkhead domain, respectively (Figure 1). All mutations were classified as pathogenic variants according to the American College of Medical Genetics (ACMG) genomics standards and guidelines for the interpretation of sequence variants (23). The mutation of case 1 c.763T>C (p.Trp255Arg) had never been reported before.

**Table 1:** Clinical Features of Subjects with FOXG1-related syndrome.

	**Case 1**	**Case 2**	**Case 3**
Gene mutation	c.763 T>C	c. 250delC	c.256dupC
Amino acid change	(p.Trp255Arg)	(p.Gln86Argfs*106),	(p.Gln86Aspfs*34)
Age of diagnosis (years)	1.92	15.91	1.67
Age (years)	2.58	17.33	1.83
Head circumference (cm), (percentile)	42 (<3rd)	49 (<3rd)	45 (<3rd)
Gross motor development			
Held upright, holds head steady	+	+	+
Rotates head	+	+	+
Move arms and legs vigorously	+	+	+
Forearm supported (prone position)	+	+	+
Rolls over	+	–	+
Sits supported, head steady	+	+	+
Stands with hands help	+	–	–
Epilepsy	+	+	+
Seizure type	Focal	Generalized, focal	Focal (FLE)
Onset (years)	2.6	1	1.5
EEG			
Epileptiform discharge	–	Right fronto-central, left centro-pareito-temporal	Cz
Slowing of background	+	+	–
Hypotonia	+	–	+
Respiratory arrhythmia	–	–	–
Impaired social contact	+	+	+
Movement disorders			
Stereotypies	+	+	+
Dystonia	+	+	+
Rigidity	–	+	–
Chorea-athetosis	+	–	+
Orofacial-dyskinesia	+	+	+
Jerky limb movement	+	+	+
Brain MRI			
Dysgenesis of corpus callosum	+	+	+
Underdevelopment of frontal areas	+	+	+
Hypomyelination	+ (Temporal)	–	+ (Frontal, Internal capsule)

**Figure 2:**
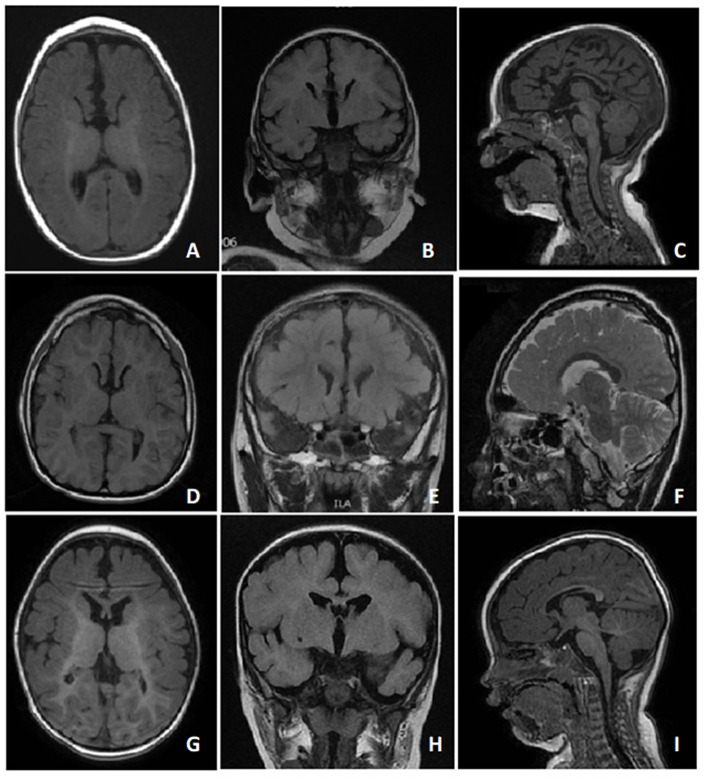
T1 and T2 MRI images of case 1 **(A-C)** at 6 months of age, case 2 **(D-F)** at 7 years of age, and case 3 **(G-I)** at 11 months of age showed dysgenesis of the corpus callosum, including the genu and body portions of the corpus callosum.

All patients had microcephaly with developmental delay found at around 2 to 4 months of age (Figure 2). Early-onset (<1 years of age) hyperkinetic movement disorders with predominantly orofacial dyskinesia and jerky limb movements appeared first, and then stereotypies, mostly hand wringing, developed later. The movement disorders evolved with age in case 2, who was 17 years of age at time of writing. The hand stereotypies and chorea-athetosis with jerky movements, which appeared at early childhood, have decreased markedly. In contrast, prominent dystonia and rigidity developed over his trunk and extremities.

The MSEL evaluation results (Table 2) showed that our three patients had severe psychomotor impairments, with DQ < 55 in all MSEL scales, in particular, case 2 who was 17 years of age had all DQs < 5. The gross motor and expressive language scales were more severely affected than the other scales in all the patients, with lower DQs and an overall mental age of≤6 months. We found uneven cognitive abilities in verbal and non-verbal cognitive domains in all of them, with ~2 month differences. Cases 1 and 3 had better non-verbal than verbal abilities, while the non-verbal abilities were worse than verbal abilities in Case 2, who was in his adolescence.

**Table 2:** The evaluation of Mullen Scales of Early Learning (MSEL) of Subjects with FOXG1-related syndrome.

	**Case 1**	**Case 2**	**Case 3**
**Biological Age at evaluation (months)**	27	203	23
**Age equivalent (months)**			
Overall mental age	9	5.25	6.5
Non-verbal mental age	10	4.5	7.5
Verbal mental age	8	6	5.5
**Subscales**			
Visual reception	11	6	8
Fine motor	9	3	7
Receptive language	11	7	5
Expressive language	5	5	6
Gross motor	6	3	5
**Developmental quotient (DQ)**			
Overall cognition	33.3	2.6	28.3
Nonverbal	37.0	2.2	32.6
Verbal	29.6	3.0	23.9
**Subscales**			
Visual reception	40.7	3.0	34.8
Fine motor	33.3	1.5	30.4
Receptive language	40.7	3.5	21.7
Expressive language	18.5	2.5	26.1
Gross motor	22.2	1.5	21.7

The developmental performance evaluated by MSEL demonstrated non-verbal (Table 3) and verbal (Table 4) performances among our three cases. Regarding non-verbal performance, Case 1 had the highest developmental performance, whereas Case 2 had the lowest performance. All three cases showed visual abilities to horizontal and far/near localized tracking, look and track for dropping items, and cord pull to obtain items. In addition, all of them performed basic hand functions, such as holding reflex and grasping six-inch pegs without using their thumbs. Cases 1 and 3 had comparable non-verbal performances, except that Case 1 was able to match objects without naming them and could pincer-grasp and bang blocks in the midline, while Case 3 could not. Both Cases 1 and 3 were able to play with a ball, attend to pictures, and grasp blocks with their thumbs and drop them, while Case 2 lacked those skills.

**Table 3:** Developmental performance of MSEL nonverbal skills in the subjects with FOXG1-related syndrome.

**MSEL nonverbal skills items**	**Case 1**	**Case 2**	**Case 3**
Visual tracking (Horizontal)	180 degrees	180 degrees	180 degrees
Localization	1. Horizontal shift 2. Far and near	1. Horizontal shift 2. Far and near	1. Horizontal shift 2. Far and near
Star at own hand	Yes	Yes	Yes
Look for dropping spoon	Yes	Yes	Yes
Pull cord to obtain	Yes	Yes	Yes
Turn cup upside down	No	No	No
Make object association	Ball	No	Ball
Attend to pictures	Yes	No	Yes
Match objects without naming	Shoes, Cars, Keys, Sticks	No	No
Arm and hand reflex	1. Hold ring reflexively 2. Midline 3. Grasp reflex integrated	1. Hold ring reflexively 2. Midline	1. Hold ring reflexively 2. Midline 3. Grasp reflex integrated
Grasp	1.6 inches peg (ulnar palmar) 2. Block (with thumb) 3. Drop the block	6 inches peg (ulnar palmar)	1.6 inches peg (ulnar palmar) 2. Block (with thumb) 3. Transfer or drop the block
Pincer Grasp and Bang	1. Refined pincer grasp 2. Bang the blocks in midline	No	No

**Table 4:** Developmental performance of MSEL verbal skills in the subjects with FOXG1-related syndrome.

**MSEL verbal skills items**	**Case 1**	**Case 2**	**Case 3**
Sound response	1. Alert to sound 2. Social smiling and vocalizing	1. Alert to sound 2. Social smiling and vocalizing	1. Alert to sound 2. Social smiling and vocalizing
Listening coordination	With turning and looking	With turning and looking	With turning and looking
Enjoy self mirror interaction	Yes	No	Yes
Attend to words and movement	Yes	Yes	No
Language recognition	Familiar names and words, and own name	Familiar names and words, and own name	No
Language understanding	Inhibitory words, simple verbal input, and command with gesture	No	No
Smiling and laughing	Make more than one kind of laughter	Make more than one kind of laughter	Make more than one kind of laughter
Vocalization	1. Make “ah, eh, m” 2. Play with own sounds	1. Make “ah, eh, m” 2. Play with own sounds	1. Make “ah, eh, m” 2. Play with own sounds 3. Voluntary babbling

Case 1 had the highest developmental verbal performance, whereas Case 3 had the lowest. All cases were able to respond to sounds by smiling and laughing and could listen attentively by turning their heads and looking when the examiner made a sound. In addition, all of them could make more than one laughter and play with their own sounds. Cases 1 and 3 enjoyed self-mirror interactions, whereas Case 2 did not. Although Case 3 had the lowest verbal performance, he was the only one who could babble voluntarily. Compared with Case 3, both Cases 1 and 2 were capable of attending to words and movement, and recognized familiar names, words, and their own names. Moreover, Case 1 could even understand inhibitory words, simple verbal inputs (i.e., bye-bye or clap), and gesture commands.

## Discussion

We report the first case series of *FOXG1*-related syndrome in Han Chinese children in Taiwan. We found two novel mutations that were never described before. This is the first study showing the clinical progression of movement disorders overtime in patients with *FOXG1*-related syndrome. The movement disorders changed from hyperkinetic in early childhood to more hypokinetic in adolescence, along with the development of dystonia.

*FOXG1*, formerly BF-1, is a transcription repressor expressed in both fetal and adult brains and is composed of one coding exon and belongs to the forkhead (FOX) family of genes that were identified in animals ranging from worms to humans. It is essential for the development of the forebrain (telencephalon) and for structures derived from the telencephalon, including the cerebral cortex, hippocampus, and basal ganglia, as shown in animal models (24, 25).

The *FOXG1*-related syndrome was initially described as a congenital variant of the RTT (2–4), but phenotypic studies found different features from it, like the presence of hyperkinetic movements in early infancy and characteristic brain imaging abnormalities, as well as the lack of regression and lack of respiratory arrhythmias (5–8, 11).

In line with other studies (5–8, 11), all our three cases demonstrated cardinal symptoms of *FOXG1*-related syndrome. They all developed acquired microcephaly at around 2 to 4 months of age. The early-onset hyperkinetic movement disorders with developmental delays were the first symptoms noted. The stereotypies developed later with characteristic brain MRI findings including dysgenesis of the corpus callosum over the genu and body (5, 6). All our cases also presented underdevelopment of the frontal areas of the cerebrum.

To the best of our knowledge, no long-term evaluations of movement disorders in *FOXG1*-related syndrome have been reported. In our series, Case 2 presented with hyperkinetic movement disorders, including prominent orofacial dyskinesia, jerky limb movements, and choreoathetosis in early stage. However, he developed limb rigidity and dystonia with less hand stereotypies when getting older. Therefore, *FOXG1*-related syndrome should still be considered in older children or adults with characteristic brain image findings, acquired microcephaly, and hypokinetic movement disorders, even if lacking hyperkinetic movements. However, because this is only a single case demonstration, more data from adolescent and adult patients with *FOXG1*-related disorder are needed to clarify the evolution of movement disorder.

After assessing the clinical severity of various phenotypes including somatic growth, motor and speech development, behavior, neurological features, and MRI anomalies, Mitter et al. suggested that subjects with frameshift or non-sense variants in the N-terminal domain and forkhead domain (except for conserved site 1) had more severe phenotypes than those with missense variants in the forkhead conserved site 1 (8). However, no neuropsychological evaluation was done in that study.

In our study, alterations of base pair 256 in the N-terminal domains in Cases 2 and 3 both harbored mutations of c.256delC (p.Gln86Argfs^*^106) and c.256dupC (p.Gln86Aspfs^*^34), respectively (Figure 1), generating truncated proteins that completely or partially lack the forkhead domain. In line with the study of Mitter et al. (8), both showed severe phenotypes with severe psychomotor impairment, and their verbal and non-verbal mental age were between 4.5 and 8 months (Table 1). In contrast, Case 1, who had a novel missense mutation c.763T>C (p.Trp255Arg) located in the forkhead domain, had better cognitive abilities. Interestingly, Case 2, the oldest subject (17 years old), had the worst cognitive abilities among them, particularly in fine and gross motor domain. These findings may be explained by the progressive extremity rigidity with age limiting voluntary movements of the hands of this case.

## Conclusions

In conclusion, our cases highlighted the uneven verbal and non-verbal cognitive abilities of subjects with *FOXG1*-related syndrome. Of note, individuals with *FOXG1*-related syndrome may show clinical progression from hyperkinetic to hypokinetic features with age. Therefore, the *FOXG1* mutation should be considered in individuals with a history of hyperkinetic movements in childhood, stereotypies, microcephaly, and characteristic brain images. However, our findings are limited by small case number. Therefore, future studies which enroll more cases are needed to validate this finding.

## Data Availability

The datasets for this manuscript are not publicly available because all data had been shown in the paper. Requests to access the datasets should be directed to leeped@hotmail.com.

## Ethics Statement

This study was carried out in accordance with the recommendations of the Ethical Committee of National Taiwan University Hospital with written informed consent from all subjects. All subjects gave written informed consent in accordance with the Declaration of Helsinki. The protocol was approved by the Ethical Committee of National Taiwan University Hospital.

## Author Contributions

L-CW contributed to the study design, data collection, and writing. Y-TW, C-JH, W-CW, and W-CT contributed to data collection. W-TL contributed to the study design and conduct, writing, and revision.

### Conflict of Interest Statement

The authors declare that the research was conducted in the absence of any commercial or financial relationships that could be construed as a potential conflict of interest.
